# Induction of labor after one previous Cesarean section in women with an unfavorable cervix: A retrospective cohort study

**DOI:** 10.1371/journal.pone.0200024

**Published:** 2018-07-02

**Authors:** Tove Wallstrom, Jenny Bjorklund, Joanna Frykman, Hans Jarnbert-Pettersson, Helena Akerud, Elisabeth Darj, Kristina Gemzell-Danielsson, Eva Wiberg-Itzel

**Affiliations:** 1 Department of Clinical Science and Education Karolinska Institute, Women’s Clinic, Sodersjukhuset, Sweden; 2 Department of Immunology, Genetics and Pathology, Uppsala University, Uppsala, Sweden; 3 Department of Public Health and Nursing, NTNU, Norwegian University of Science and Technology, Oslo, Norway; 4 Department of Women’s and Children’s Health, Karolinska Institutet, Karolinska University Hospital, Stockholm, Sweden; Federal University of Sergipe, BRAZIL

## Abstract

**Objective:**

Uterine rupture is a well-known but unusual complication in vaginal deliveries with a Cesarean section in the history. The risk of uterine rupture is at least two-fold when labor is induced. In Sweden, women are allowed to deliver vaginally after one previous Cesarean section, regardless if labor starts spontaneously or is induced. The aim of the study is to compare the proportion of uterine ruptures between the three methods (balloon catheter, Minprostin® and Cytotec®) for induction of labor in women with an unfavorable cervix and one previous Cesarean section.

**Material and methods:**

Retrospective cohort study of all women with one previous Cesarean section and induction of labor with an unfavorable cervix at the four largest clinics in Stockholm during 2012–2015. Inclusion criteria: Women with a previous Cesarean section and induction of labor with a viable fetus, cephalic presentation, singleton, at ≥34 w, (n = 910).

**Results:**

3.0% **(**27/910) of the women with induction of labor had a uterine rupture, 91% of them had no previous vaginal delivery. The proportion of uterine ruptures was 2.0% (6/295) with orally administrated Cytotec®, 2.1% (7/335) with balloon catheter and 5.0% (14/ 281) when Minprostin® was used.

**Conclusions:**

No difference in the proportion of uterine ruptures was shown when orally administrated Cytotec® and balloon catheter were compared (p = 0.64). Orally administrated Cytotec® and balloon catheter give a high success rate of vaginal deliveries (almost 70%) despite an unfavorable cervix.

## Introduction

Cesarean section (CS) is a common procedure in obstetric care, with 25–50% of all deliveries worldwide ending with a CS[1]. A previous CS is known to be one of the strongest risk factors for uterine rupture (UR) in the following delivery [2–4]. The incidence of UR among women with one previous CS is assumed to be 0.5–0.9% [2, 3, 5], compared to 0.2 ‰ in women without any history of CS [4]. Factors such as induction of labor (IOL), fetus large for gestational age (> 4000g), labor dystocia, maternal height ≤ 160 cm and age > 35years are all known to increase the risk of a UR [4–7]. Although in women with a previous CS the overall incidence of UR is low. Women with a vaginal delivery before or after the primary CS are more likely to have a successful vaginal delivery even after a previous CS in the subsequent labor [8–11].

IOL after a previous CS is a controversial intervention. The proportion of UR in the Trial of Labor after Cesarean section group (TOLAC) with IOL is reported as high as 1.4% and an association with the use of vaginal prostaglandins has been shown [2, 3, 12–15].

Misoprostol (Cytotec®) is a prostaglandin, used for IOL worldwide. Since 2012, Cytotec®, administrated as an oral solution, is the recommended method for IOL in Sweden. From the onset, this was also used among women with one previous CS.

At Sodersjukhuset in Stockholm the proportion of subsequent CS decreased from on average 40.5% before the use of oral solution of Cytotec® to 23.0% afterwards. Before Cytotec®, Minprostin® 1mg and balloon catheter were used as primary methods for IOL in this group of women. There was no increase in maternal or fetal complications after the change of method (only statistics from medical records is available, no published data.)

In Sweden, IOL is recommended after one previous CS. After 2016 Cytotec® was discouraged in the Swedish guidelines for IOL after one previous CS, even though scientific knowledge was limited and based only on small studies where only vaginal and not oral administrated solution of Cytotec® was used [1, 16–18].

No prostaglandins at all were recommended as they were considered to increase the risk of UR, compared to balloon catheter[3, 13].

*The Primary aim of this study was to compare* the proportion of UR between the three methods (balloon catheter, Minprostin® and Cytotec®) for IOL in women with an unfavorable cervix and one previous CS. The secondary endpoint was to study the delivery outcomes such as mode of delivery and the outcome of the new-born in the TOLAC group.

## Material and methods

This was a retrospective cohort study of all women with one previous CS, unfavorable cervix, and IOL at the four largest hospitals in Stockholm: Danderyd’s hospital (no 1), Karolinska Hospital in Huddinge (no 2), Karolinska Hospital in Solna (no 3), and Sodersjukhuset (no 4) during the period from 2012 to 2015 (n = 910). Data from all induced deliveries at these four delivery wards during the actual study period were collected from the Obstetrix database (the main used medical record system in Sweden) All personal data were encoded, so that individuals could not be identified in the analysis.

*Inclusion criteria for the study* were women with one previous CS and IOL. All women included had a viable fetus in cephalic presentation, singleton, at gestational age of ≥34 weeks.

Different types of medical preparations such as vaginal dinoproston (Minprostin®, a vaginal gel, Pfizer, NY, USA, or Propess®, an insert with slow-release of prostaglandin E2, Ferring, Malmo, Sweden) or mechanical methods (balloon catheters, Bard®/Rusch®, Cook Medical, USA) were used previously for IOL as primary methods in the case of an unfavourable cervix (Bishop score (BS) ≤5). From 2012, Cytotec® was also administrated as an oral solution for the same indication at the included hospitals.

*In the Cytotec® group*, Cytotec® was prepared as an oral solution, one tablet of 200 μg Cytotec® was dissolved in 20 ml of water. The solution thus held 10 μg of Cytotec®/ml. This method of administration has been tested by the Swedish Institute of Pharmacology [19] and approved to be accurate in terms of correct dosage. A dose of 2.5 ml / 25 μg of Cytotec® were administered orally to the women every two hours until frequent painful contractions were obtained. The dose could be repeated up to eight times if necessary (maximum 200ug in 24h). When ripening of the cervix had been achieved (BS ≥ 5), amniotomy and oxytocin were used to support uterine contractions.

*In the Minprostin® group*, 1 or 2 mg of Minprostin® was given vaginally. The progress of induction was evaluated by a vaginal examination every six hours. In the case of a still unfavourable cervix, additional doses were administered, up to a total of three doses (6 mg). When ripening of the cervix had been achieved (BS ≥ 5), amniotomy and oxytocin were used to support labor contractions.

*In the balloon group*, mechanical IOL was performed with a balloon catheter (Bard®). The balloon was inserted into the cervix beyond the internal os and the bulb inflated with 50 ml of sterile water. The midwife stretches the catheter every 30 minutes. The balloon could be used for a maximum of 10h. At a cervical dilation of at least 3 cm, the balloon was expelled and the induction was followed by amniotomy and oxytocin.

No additional method was used after the primary method of induction in 84.1% (248/295) deliveries in the Cytotec® group, in 80.4% (226/281) in the Minprostin® group and in 97.1% (326/335) in the balloon group. As most of the women (minimum 80%) received only one method of induction, we consider it appropriate to use the first method of induction as the main method for calculation. 37.5% (105/280) of the women in the Minprostin® group received two doses of Minprostin® and 9.3% (26/280) received a third dose.

A total UR was defined as a complete separation of all layers of the uterine wall, including myometrium and the serosa. A dehiscence was defined as a separation of the muscular layers, with intact serosa[20]

IOL in an unfavorable cervix strives to reach amniotomy. Amniotomy itself is therefore not considered as an additional method of IOL in this study. Amniotomy followed by stimulation with oxytocin were used in deliveries with a favourable cervix (BS ≥ 6), and oxytocin only, was used primarily in cases with ruptured membranes and a favourable cervix.

Immediately after birth, before the new-born first cry, an arterial and a venous cord blood sample were drawn from a segment of the cord without clamping. Arterial pH and base deficit (BD) were analyzed within a few minutes with a point-of-care device (ABL 800 Bayer®), available in the delivery wards. This is a routine clinical procedure in all the delivery wards in Stockholm.

### Statistics

One-way ANOVA (ANalysis Of Variance) was used to compare mean values, and the Chi-square test to compare the proportions, between the different methods of induction. In this study the association between the proportion of UR (primary outcome) and method of induction was analysed, and logistic regression was used to adjust this association with respect to other risk factors for UR. We adjusted for possible explanatory variables such as maternal age (<30 years or >30 years), previous vaginal delivery (yes or no), gestational age (<41+0 weeks or ≥41+0 weeks), delivery unit/hospital (no 1–4), indication for induction (Premature Rupture Of Membrane (PROM), Postdate (≥41 weeks), maternal, fetal, non-medical, prolonged latent phase), BS (≤5 or >5), method of induction (Cytotec®, Minprostin®, balloon), delivery time (≤12h, >12≤24h, >24h), time of augmentation with oxytocin (<10h, >10h) and type of CS (Elective Cesarean section (ES), Acute Cesarean section (AS)). First, we calculated the crude (unadjusted) associations of each possible explanatory variable and UR. Second, we used multivariable models to study the adjusted associations with respect to the variables above. Finally, to study whether the method of induction differed in any subgroup with respect to the levels of variables, we added an interaction to the adjusted model between the method of induction and each of the possible explanatory variable, one at a time. Model fit was judged based on the Hosmer and Lemeshow Goodness-of-fit test, if p>0.05 the model fit was acceptable. The associations are presented as odds ratios (OR) with 95% confidence intervals (CI).

No pre-power calculation was done since the study was register based and the rationale for the number of observations therefore was to use all available data. However, we performed a post-hoc calculation to see how differences in our primary outcome (rupture (yes/no)) that the study was dimensioned to detect with the actual number of individuals. We would have 80% power (5% significance level, two-sided) to detect a difference in the proportion with uterine rupture of 2.0% vs 7.3% between the groups of women who received Cytotec® (n = 295) and Minprostin® (n = 280). A p-value <0.05 was considered statistically significant. Statistical analyses were performed using SPSS 23.0 (SPSS Inc., Chicago, IL.).

### Ethical approval

The study was approved by the regional ethics committee (Karolinska Institute, file record: 2016/1494). The study complies with the World Medical Association Helsinki Declaration regarding ethical conduct of research involving human subjects.

## Results

**Fig 1:**
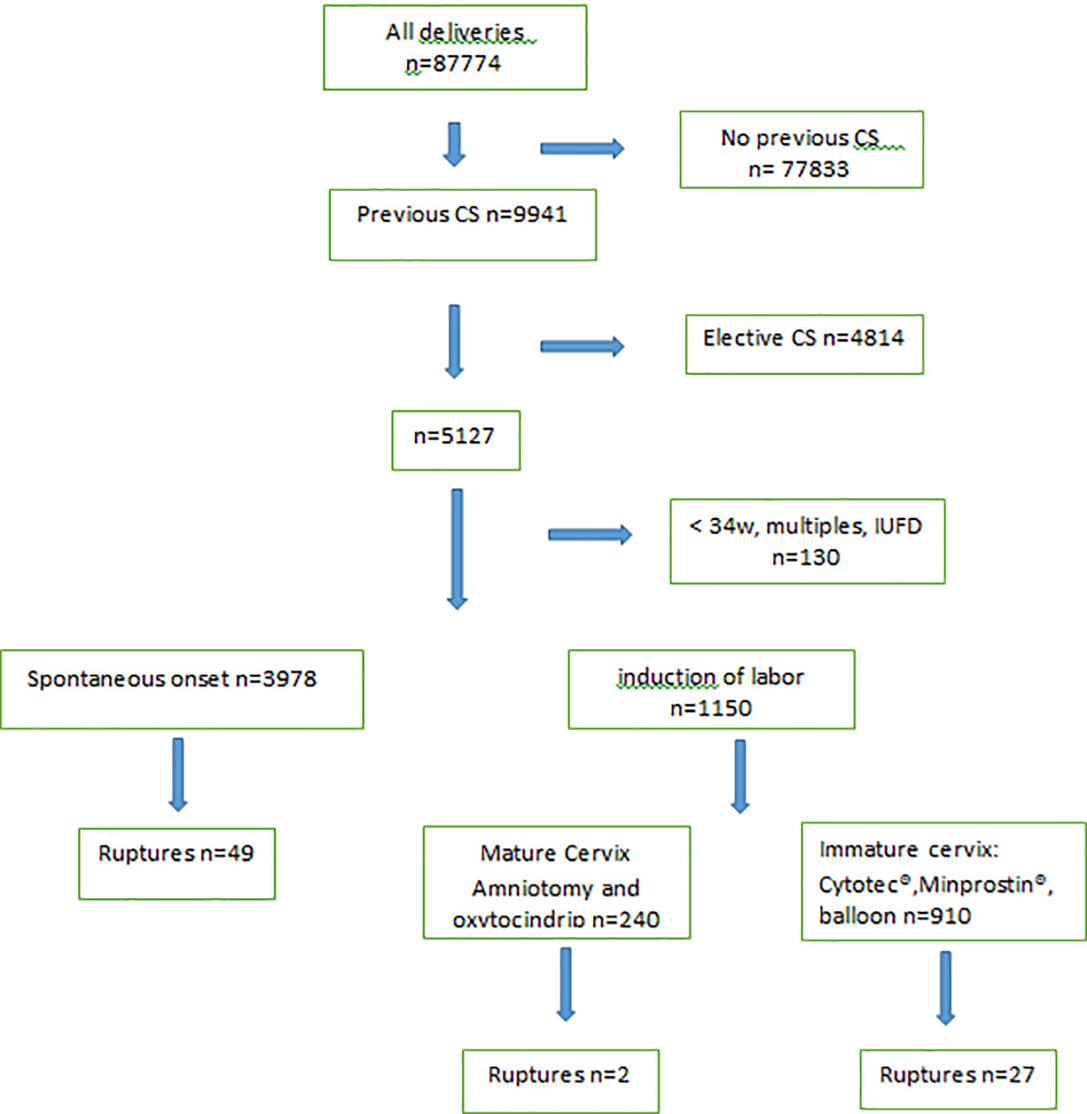
Flowchart.

During study time 87,774 deliveries occurred at the four largest delivery wards in Stockholm. There were 11.3% (9,941/87,774) women that had a previous CS, and almost every second of them (48.4%) were planned for an ES before onset of labor. After exclusion of pregnancies <34 gestational weeks, multiples and IUFD, 4,997 deliveries remained. There were 23.0% (1,150/4,997) of the remaining deliveries that went for IOL. The majority, 79% (910/1,150) of them had an unfavorable cervix with a BS≤5, and a balloon catheter, Minprostin® or an oral solution of Cytotec® were used for induction of labor (Fig 1).

Table 1, describes baseline data from all the induced deliveries with an unfavorable cervix with respect to the method of induction. No statistically significant difference was found between the three groups of inductions except maternal age, where women in the balloon group were younger (32.5 years) compared to the Minprostin® group (33.4 years) and Cytotec® group ((34.1 years) p<0.01). There was a significant difference between methods of choice between the four hospitals in Stockholm (p<0.01). Cytotec® was the method of choice at hospital no 4 (89.5%), Minprostin® at hospital no 1 and no 2 (44.6% vs 37.9%), and a balloon catheter primary at hospital no 3 (47.8%, Table 1).

**Table 1:** Maternal baseline data, indications for previous CS, indications for IOL and BS among women with IOL after a previous CS and an unfavourable cervix, presented per method of induction (n = 910). Data are presented as numbers (%) or mean (Standard deviation (SD)). P -Values<0.05 were considered as statistically significant*.

	Cytotec® (n = 295)	Minprostin® (n = 280)	Balloon (n = 335)	p-value
**Hospital**				<0.01*
No 1 (n = 206)	3 (1.0)	125 (44.6)	78 (23.3)	
No 2 (n = 211)	14 (4.7)	106 (37.9)	91 (27.2)	
No 3 (n = 222)	14 (4.7)	48 (17.1)	160 (47.8)	
No 4 (n = 271)	264 (89.5)	1 (0.4)	6 (0.2)	
**Age (years)**	34.1 (4.9)	33.4 (4.9)	32.5 (5.3)	<0.01*
**BMI (kg/m2)**	25.7(5.1)	26.7(5.1)	26.0(5.2)	0.052
**BP (mm Hg)**	123/79 (14.5/10.8)	121/75 (15.6/11,1)	122/77 (15.5/11.4)	0.21/ <0.01*
**No prev. vag. del (%)**	222 (75.3)	211 (75.4)	258(77.0)	0.84
**Indication for previous CS**				0.55
dystocia (n = 297)	70 (23.7)	81 (28.8)	96 (28.7)	
fetal distress (n = 293)	74 (25.1)	74 (26.3)	77 (23.1)	
breech/transverse (n = 254)	77 (26.1)	56 (19.9)	67 (20.1)	
maternal (n = 117)	32 (10.8)	26 (9.3)	34 (10.2)	
fetal (n = 81)	18 (6.1)	22 (7.8)	23 (6.9)	
ablatio / previa (n = 55)	10 (3.4)	11 (3.9)	19 (5.7)	
unknown (n = 31)	9 (3.1)	7 (2.5)	7 (2.1)	
women request (n = 22)	5 (1.7)	4 (1.4)	11 (3.3)	
**Previous AS (n = 673)**	205 (69.5)	215 (76.8)	253 (75.5)	0.12
**Gest. age (days)**	281 (12.1)	282 (12.3)	281 (11.6)	0.50
***Indications for induction***				P<0.01*
Postdate pregnancies (n = 225)	75 (25.4)	83 (29.5)	67 (20.0)	
PROM (n = 195)	69 (23.4)	49 (17.8)	77 (23.1)	
Maternal reason (n = 128)	38 (12.9)	43 (15.3)	47 (14.1)	
Fetal reason (n = 177)	53 (18.0)	63 (22.4)	61 (18.3)	
Non-medical reason (n = 137)	35 (11.9)	40 (14.2)	62 (18.6)	
Prolonged latent phase (n = 48)	25 (8.5)	2 (0.7)	21 (6.3)	
**BS (0–10)**	2.9 (1.7)	2.4 (1.6)	4.0 (1.5)	<0.01*

A statistically significant difference in BS at the time of induction was found between the three groups, with highest BS (4) in the balloon group and the lowest (2.4) score in the Minprostin® group (Table 1.).

There was a significant difference in the indication for induction (p<0.01). The most common reason overall was post-dated pregnancies (≥41w of gestation, 24.7%) followed by PROM (21.4%), fetal reason (19.5%), non-medical reason (15.1%), maternal reason (14.1%) and prolonged latent phase (5.7%, Table 1.).

A significant difference (p<0.01) in time from the start of induction to delivery was found among the deliveries included. The shortest time was found in the balloon group 14.3 h (1.2–43.1h) and longest in the Minprostin® group 21.7h (2.1–62.1h, Table 2.). Even a statistically significant difference in the use of oxytocin was found. The highest proportion and longest time of stimulation were found in the balloon group (88.4% and 8.5 h), and the lowest proportion and shortest time of stimulation were found in the Cytotec® group, (56.9% and 6.9 h, p<0.01, Table 2.).

**Table 2:** Labor and fetal outcome data, among women with IOL after one previous CS and an unfavourable cervix, presented per method of induction, (n = 910). Data are presented as numbers (%) or mean (SD). P -Values<0.05 were considered as statistically significant *.

	Cytotec® (n = 295)	Minprostin® (n = 281)	Balloon (n = 335)	p-value*
**Time from induction to delivery (h)**	20.4(10.8)	21.7(11.2)	14.3(6.3)	<0.01*
**Frequency of oxytocin drip (n = 637)**	168 (56.9)	173(61.8)	296(88.4)	<0.01*
**Time of Oxytocin drip(h)**	6.9(6.8)	8.2(7.1)	8.5(5.4)	0.02*
**Frequency of UR (n = 27)**	6 (2.0)	14 (5.0)	7 (2.1)	0.055
**Type of UR (n = 27)**				0.86
-Dehiscence (n = 9)	3 (50.0)	3 (21.4)	3 (42.9)	
<5cm (n = 6)	1 (16.7)	3 (21.4)	2 (28.6)	
.>5cm (n = 6)	1 (16.7)	4 (28.6)	1 (14.3)	
Total rupture (n = 6)	1 (16.7)	4 (28.6)	1 (14.3)	
**Way of delivery (%)**				0.02*
Vaginal (n = 595)	204 (69.2)	160 (57.1)	231 (69.0)	
CS (n = 315)	91 (30.8)	120 (42.9)	104 (31.0)	
**PPH**				
>1000ml (n = 115)	40 (13.6)	34 (12.1)	41 (12.2)	0.85
>1500ml (n = 56)	25(8.5)	14 (5.0)	7 (2.1)	0.13
**Fetal weight (g)**	3587.1 (563.0)	3577.6 (564.4)	3551.8 (564.2)	0.72
**Fetal height (cm)**	50.7(2.3)	50.7(2.3)	50.7(2.4)	0.81
**Fetal head circumference (cm)**	35.1 (1.6)	35.3 (1.6)	35.1 (1.5)	0.24
**Apgar <7, 5’**	3 (1.0)	6 (2.2)	4 (1.2)	0.47
**pH < 7.10 (n = 41)**	16(6.8)	11(5.5)	14(5.6)	0.15
**Missing (n = 222)**	58(19.7)	80(28.6)	84(25.1)	
**Fetal death (n = 0)**	0	0	0	1.0

The mode of delivery varied between the three different groups presented. CS was most common in the Minprostin® group (42.9%), compared to almost similar frequencies in the balloon (31.0%) and the Cytotec® (30.8%) groups (Table 2).

Three percent (27/910) of the deliveries included had a UR discovered during or after delivery. A difference among deliveries with UR was shown according to the proportion of a previous vaginal delivery. The majority (91%) of the women with UR had no history of vaginal delivery, compared to 69% of the women with no UR (p<0.01).

In 22.2% (6/27) the UR was considered as total (at least a part of the fetus in the abdominal cavity), and in 33.3% (9/27) a dehiscence was found. No difference was identified among the groups according to the size of the rupture (5< or ≥5cm), 22% (6/27) had a UR <5cm and the same proportion had a UR >5 cm. No statistically significant difference was found according to the method of induction and type of rupture (Table 2).

When studying the method of induction, it can be concluded that 5.0% (14/280) of the UR occurred in the group where Minprostin® was used, 2.1% in the balloon group (7/335) and 2.0% (6/295) in the group with orally administrated Cytotec® (6/295). The remaining cases were found in the group where amniotomy was used as the primary method of IOL (2/190, 1.1%).

No statistical difference was found when considering the indications for IOL. There was neither a statistical difference between those women who had a UR and those who did not, regarding the status of the cervix at the beginning of the induction (Table 3).

**Table 3:** Associations between possible risk factors for uterine rupture (UR) and UR in induction of labor in an immature cervix. Values are expressed as Odds Ratio (OR) with corresponding 95% confidence intervals (CI). N = 910. P -Values<0.05 were considered as statistically significant*.

Risk factors for UR after induction of labor in an immature cervix.	UR /total (%)	OR unadjusted (95% CI)	OR adjusted (95% CI)
**Maternal Age (years)**			
< 30	9/248 (3.6)	Ref.	Ref.
≥ 30	18/662 (2.7)	0.7 (0.3–1.7)	0.7 (0.3–1.7)
**Parity**			
Previous vag. delivery	2/219 (0.9)	Ref.	Ref.
No previous vag. delivery	25/691 (3.6)	4.1 (1.0–17.3) *	2.8 (0.6–12.6)
**Gestational age (w)**			
< 41+0	14/548 (3.1)	Ref.	Ref.
≥ 41+0	13/362 (3.6)	1.4 (0.7–3.1)	1.0 (0.4–2.6)
**Type of previous CS**			
ES (Elective)	9/236 (3.8)	Ref.	Ref.
AS (Acute)	18/674 (2.7)	0.7 (0.3–1.5)	0.7 (0.2–1.9)
**Hospital**			
No 4	6/271 (2.2)	Ref.	Ref.
No 3	4/222 (1.8)	1.5 (0.5–4.6)	0.7 (0.05–11.3)
No 2	7/211 (3.3)	0.8 (0.2–2.9)	0.4 (0.03–6.5)
No 1	10/206 (4.9)	2.3 (0.8–6.3)	0.8 (0.1–12.5)
**Indication for induction**			
PROM	7/195 (3.6)	Ref.	Ref.
Postdate (≥ 41)	10/225 (4.4)	1.3 (0.5–3.3)	1.1 (0.4–3.7)
Maternal	3/128 (2.3)	0.6 (0.2–2.5)	0.6 (0.1–2.5)
Fetal	3/177 (1.7)	0.5 (0.1–1.8)	0.5 (0.1–2.0)
Non-medical	2/137 (1.5)	0.4 (0.1–1.9)	0.4 (0.1–2.2)
Prolonged latent phase	2/48 (4.2)	1.2 (0.2–5.8)	1.8 (0.3–9.7)
**Bishop score**			
>5	1/76 (1.3)	Ref.	Ref.
≤5	26/834 (3.1)	2.4 (0.3–18.0)	0.4 (0.05–3.2)
**Method of induction**			
Balloon catheter	7/335 (2.1)	Ref.	Ref.
Cytotec®	6/295 (2.0)	1.0 (0.3–2.9)	0.3 (0.02–4.9)
Minprostin®	14/280 (5.0)	2.5 (1.0–6.2) *	1.2 (0.4–3.9)
**Delivery time (h)**			
≤ 12	4/254 (1.6)	Ref.	Ref.
> 12 - <24	10/433 (2.3)	1.5 (0.5–4.8)	1.3 (0.4–4.4)
>24	13/223 (5.8)	3.9 (1.2–12.0) *	3.4 (0.9–13.0)
**No Oxytocin drip**	7/273 (2.6)	Ref.	Ref.
**Oxytocin drip**	20/637 (3.1)	1.2 (0.5–3.1)	1.1 (0.4–2.9)
**Time of oxy. drip (h)**			
<10h	21/736 (2.9)	Ref.	Ref.
>10h	6/174 (3.4)	1.2 (0.5–3.1)	0.7 (0.3–2.0)
**Type of previous CS**			
ES (Elective)	9/236 (3.8)	Ref.	Ref.
AS (Acute)	18/674 (2.7)	0.7 (0.3–1.5)	0.7 (0.3–1.6)

No previous vaginal delivery was the strongest risk factor in this study for having a UR during labor (OR 4.1; 95% CI; 1. -17.3, Table 3) after one previous CS, but after adjustment for the other possible risk factors for UR the result was no longer statistically significant (aOR 2.8; 95% CI; 0.6–12.6). Prolonged labor >24 h was associated with an increased risk for having a UR (OR 3.9; 95% CI; 1.2–12). After adjustment for the other factors, the result was no longer significant (aOR 3.4 95% CI; 0.9–13.0).

Induction of labor with Minprostin® was associated with an increased risk for having a UR (OR 2.5.; 95% CI; 1.0–6.2, Table 3.). After adjustment for other possible risk factors for UR in Table 3, the result was no longer significant (aOR 1.2; 95% CI; 0.4–3.9).

No significant interactions were found (p-values between 0.27 and 1.0). The Hosmer and Lemeshow test and Goodness of fit test were equal to p = 0.50 for the adjusted model (n = 910).

The proportion of low Apgar score <7 after 5 min was statistically significant (11.5% vs 0.8%), low pH <7.10 (20.5% vs 4.4%) and post-partum hemorrhage (PPH)>1000 ml (30.8% vs 11.2%) between UR and non-UR groups. The proportion of CS was statically significantly higher in the UR group (79.5%) than in the non-UR group (26.8%).

## Discussion

### Main findings

Our main finding in this project is that the proportion of UR was equal when a balloon catheter was used compared to the group with orally administrated Cytotec® (2.1 vs. 2.0%, p = 1.0). The success rate of vaginal delivery after one previous CS (VBAC) in women with IOL is very high, almost 70% with both balloon catheter and oral solution of Cytotec®, regardless of BS.

### Strengths and limitations

The strengths of this project are that there are no other studies where oral solution of Cytotec® administrated in this way is presented among women with one previous CS. Even though this is a retrospective study, a careful study was made of the medical records of all women included, which make the results even more reliable.

A limitation of this project is that the study is not a randomized controlled trial, which would have been desirable. Although the study population is large, the main outcome -UR- is unusual, which means that even larger populations need to be studied. Another question is whether randomization in this group would be perceived as ethical, when there is an increased risk for UR with IOL in the group of women with a previous CS?

Another limitation in the study is that the balloon group had a significant higher BS (4) compared to Cytotec® (2.9) and Minprostin® (2.4, (p <0.001)) and therefor probably had the shortest time interval from IOL to delivery, 14.3 h (1.2–43.1h). The longest time were found in the Minprostin® group 21.7h (2.1–62.1h) where the BS was lowest. However, the rate of UR in the balloon group was similar compared to the Cytotec® group despite the shorter delivery time and more favorable cervix.

### Interpretation

Whether prostaglandins given in different forms increase the risk of UR compared to other methods of induction is a question that is still under discussion [3, 21]. According to previous studies, misoprostol administrated vaginally will increase the risk of UR [22–24]. Misoprostol in all forms has therefore been discouraged in various guidelines on labor induction in women with previous CS even though the scientific knowledge is limited [17, 18, 25]. However, the evidence is unclear and based on only small studies where only vaginally administrated misoprostol has been studied.

A solution of Cytotec® administrated orally was introduced at hospital no 4 in Stockholm at 2012 also in IOL among women with one previous CS. This was after the method had been used with very good results in the group of IOL among women without a uterine scar. The other hospitals in Stockholm introduced the method after some tests had been made by the Swedish Institute of Pharmacology [19]. The occurrence of hyper-stimulation was low (1%) in the Cytotec® group compared to the Minprostin® group where at least 15% experienced hyper-stimulation at the clinic (no published data available). Our current results are analogue to Gemzell-Danielsson et al [26, 27], How et al [28] and Aronsson et al [26] who concluded that orally administrated misoprostol actually caused less uterine hyper-stimulation than vaginal or sublingual administration. What was notable was that the proportion of CS decreased significantly in the group of women with a previous CS after IOL when an oral solution of Cytotec® was introduced at the clinic, without increasing complications.

Our study shows that IOL with oral solution of Cytotec® after one previous CS is a safe and effective method well comparable with the use of a balloon catheter, despite a more unfavorable status of the cervix. Oral solution of Cytotec® gives a significantly increased rate of VBAC compared to Minprostin® (69.2% vs 57.2%, p = 0.02) and the same successful rate as balloon catheter (69.2 vs 69.0%) even though the primary BS was different in the two groups. In our opinion, an oral solution of Cytotec® is a good alternative when IOL is needed in women with one previous CS and an unfavorable status of the cervix.

The increased risk of UR may be related to characteristics of the woman herself who receives prostaglandins rather than the drug itself; as for an example, women who receive prostaglandins are much more likely to have an unfavorable cervix than women induced with oxytocin or who enter labor spontaneously[29]. According to Harper et al [30], IOL is not associated with an increased risk of UR when duration of labor is considered. It is important to have a normal progress of labor among women with a previous CS, and all arrested deliveries should be avoided in this group. If labor is extended, a CS should be considered at an early state of labor, to avoid traumatic labor outcomes as far as possible. In our opinion it is highly important to have a various selection of methods for IOL after a previous CS especially when the cervix is unfavorable.

An important question raised in this project is whether we should continue to induce women after one previous CS. Is it justifiable that as many as 3% rupture during ongoing labor? We know that a UR during labor is often a serious event, for the woman, the fetus and for the staff in charge. Repeat CS should on the other hand be avoided if possible as they are associated with complications, but the most important criterion is to avoid the very first CS. If it is considered justifiable, the challenge might be to identify women suitable for induction, and to make the delivery as good and risk-free as possible for both the woman and her unborn child.

## Conclusion

Our final conclusions are that an orally administrated solution of Cytotec® is a good method for IOL even among women with one previous CS. Most UR in this study occurred when Minprostin® was used (5.0%), the risk is more than two-fold compared to Cytotec® and balloon catheter. No difference in the proportion of UR was shown when orally administrated Cytotec® and balloon catheter were compared (p = 0.64). Orally administrated Cytotec® as well as balloon catheter give a high success rate of VBAC (almost 70%) despite an unfavorable cervix. Our hope is that Cytotec® will be used, with caution, as one of many methods for IOL in the future.

## Supporting information

S1 Dataset Data uterine ruptures study 4. (XLSX)
